# Human Papilloma Virus Vaccination

**DOI:** 10.3390/v13061091

**Published:** 2021-06-08

**Authors:** Kendal Rosalik, Christopher Tarney, Jasmine Han

**Affiliations:** 1 Madigan Army Medical Center, Department of Obstetrics and Gynecology, Division of Gynecologic Oncology, 9040A Jackson Ave, Joint Base Lewis-McChord, WA 98431, USA; christopher.m.tarney.mil@mail.mil; 2 General Leonard Wood Army Community Hospital, Department of Obstetrics and Gynecology, 4430 Missouri Ave, Ford Leonard Wood, MO 65473, USA; jashannah@gmail.com

**Keywords:** human papilloma virus, cervical cancer, gardasil, cervarix, vaccination

## Abstract

Human papilloma virus (HPV) is the most common sexually transmitted infection worldwide causing a variety of benign and malignant conditions. A significant portion of the global population is infected with HPV, with the virus attributed to causing up to 5% of cancers worldwide. Bivalent, quadrivalent, and nine-valent vaccinations exist to aid in the prevention of these diseases and have been proven to be effective at preventing both benign and malignant disease. While vaccination is readily accessible in more developed countries, barriers exist to worldwide distribution and acceptance of vaccination. Vaccination and screening of HPV infection when used in combination are proven and predicted to decrease HPV related pathology. Improvements in vaccination formulations, for treatment as well as prevention, are actively being sought from a variety of mechanisms. Despite these advancements, and the data supporting their efficacy, there has been substantial delay in obtaining adequate vaccination coverage. In reviewing these challenges and looking forward to new vaccine development—especially within the current pandemic—it is clear from the challenges of HPV we require methods to more effectively encourage vaccination, ways to dispel vaccination myths as they occur, and implement better processes for vaccine distribution globally.

## 1. Introduction

Human papilloma virus (HPV) is a circular, non-enveloped, double stranded DNA virus belonging to the *Papillomaviridae* family of which over 200 subtypes have been identified. HPV is the most prevalent sexually transmitted infection in the world, with manifestations spanning benign and malignant processes in women and men. Pathology manifested by the virus, and therefore associated risk of benign and malignant conditions varies greatly by viral genotype, with genotypes classified as low or high-risk based on their association with malignant transformation of cells.

HPV is the most common pathogen in all female cancers, with 57,000 cancer cases in women related to HPV each year [1,2]. In men, 60,000 cancer cases per year can be attributed to HPV, with the virus causing up to 5% of all human cancers [2,3]. Worldwide, HPV-16 is thought to be most prevalent genotype followed by HPV-18, though this prevalence varies greatly by geographic region [1,4]. HPV-16 and -18 are high risk for vulvar, penile, vaginal, anal, and oropharyngeal cancers, while genotypes 6 and 11 play a role in genital warts [1,4,5]. These cancers, as detailed in Table 1, are less prevalent than cervical cancers, but many of these cancers bear high rates of morbidity and mortality when they do occur. Prevention of HPV infection, and therefore these associated conditions is an important global public health focus. While vaccination with the quadrivalent and now nine-valent vaccination has shown to be effective at preventing benign and malignant conditions, distribution of the vaccine to low- and middle-income countries has been variable. Even within countries with high access to the vaccine, uptake of vaccination has faced several challenges. While new vaccination and treatment strategies are being developed, future developments may face many if not all of the same challenges as current vaccines. Similar challenges are currently being faced in the process of vaccination against severe acute respiratory syndrome coronavirus (SARs-CoV-2), a with very clinically different presentation, but with importance to prevent infection of on a global scale. Unfortunately, there is limited information to be learned from HPV vaccination that may assist officials in instituting programs and distribution of SARs-CoV-2 vaccines; rather, advancements made on one front is likely to assist with combatting the other virus.

## 2. Benign Conditions

HPV infection can often be asymptomatic, and many will never know they were infected with 70% of women clearing infection in one year and 91% clearing the infection in two years [9]. Up to 50% of healthy people are infected with low-risk genotypes 1, 2, 4, 6, 7, 11, 27, 40–44, 54, 57, 61, 65, 72, 81, and others are linked to cutaneous warts, manifesting on a variety of body surfaces including hands and feet [1,2,17]. While HPV genotypes causing cutaneous warts varies geographically, HPV 27, 57, 2, and 1 are most common [18]. Anogenital warts are the most common clinical presentation of HPV infection worldwide and are associated with low morbidity overall [2]. These lesions can have many different appearances, being flat, raised, or cauliflower like, and vary in color [9]. Anogenital warts are highly associated with low-risk genotypes, with genotypes 6 and 11 being more common in the United States and Europe than other parts of the world [2]. The incidence of anogenital warts is increasing in children as low-risk subtypes can be transmitted non-sexually, via fomites, fingers, mouth, and skin, especially through self-inoculation or from mother to child [2,3]. These lesions are a common result of the body’s lack of ability to clear HPV infection, and while they are often asymptomatic can cause irritation, bleeding, and pain [15]. HPV infection (namely HPV-6 and HPV-11) is also responsible for benign forms of respiratory papillomatosis. While incidence of these lesions is infrequent at 3.9/100,000 adults in the United States, lesions often require retreatment and cause much distress, leading to a significant healthcare burden [19]. Benign lesions, while not as clinically serious as their counterparts, can still represent a significant burden in terms of healthcare cost, and the presence of HPV detected by deoxyribonucleic acid (DNA) testing can lead to an increase in screening, and patients may present for care regarding cutaneous warty lesions [1].

## 3. Vulvar Cancer

Vulvar cancer is an uncommon condition with an estimated 27,000 cases annually, making up 3–5% of gynecologic cancers [6]. Over 90% of vulvar cancers are squamous in nature, with HPV and immune-dependent pathways as the two separate underlying pathophysiology of cancer development, and HPV accounting for 25–43% of vulvar cancers [6]. HPV-related cancer is more slowly progressive than non-HPV related vulvar cancer [6]. HPV associated disease begins as vulvar intraepithelial neoplasia, a premalignant condition with variable appearance. Upwards of 16% of these premalignant conditions progress to squamous carcinoma of the vulva [6]. HPV-associated vulvar cancers tend to have better prognosis over non-HPV related, with a systematic review and meta-analysis completed in 2018 indicating significantly higher overall survival and disease-free survival for HPV positive disease [12,13].

## 4. Vaginal Cancer

Primary cancer of the vagina is rare, making up only 10% of cancer found within the vagina and 1–2% of female genital tract cancers [7]. Vaginal cancer has been directly associated with HPV infection and 90% of vaginal cancers are squamous in nature [7]. Precancerous lesions are identified as low-grade squamous intraepithelial lesions (LSIL) and high-grade squamous intraepithelial lesions (HSIL). LSIL can be associated with low-risk or high-risk HPV and can progress to HSIL or the infection may be cleared from the body. HSIL infections as associated with high-risk HPV genotypes. HSIL lesions have a 2–12% risk of progressing to cancer [7]. Overall 5-year survival rates of vaginal squamous cell carcinoma vary from 35–85% depending on tumor size at diagnosis and stage of disease [7,14].

## 5. Penile Cancer

Penile cancer is also rare, with an incidence of 0.8 per 100,000 in developed countries compared to 4.4 per 100,000 in developing countries, and usually impacts elderly men [8]. Similar to vulvar cancer, two pathways exist to the development of penile cancer, with the key distinction as the presence or absence of HPV. The majority of HPV infections are asymptomatic, with up to 70% clearing within one year [8]. Considered a precancerous lesion, penile intraepithelial neoplasia (PeIN) is associated with HPV and divided into categories I, II, and III, but clinically divided based off the cells from which the lesion is derived [8]. Erythroplasia of Queyrat is derived from the mucosal glans of the foreskin—with the highest risk of malignant transformation—while Bowen’s disease or squamous cell carcinoma in situ is derived from the keratinized skin of the shaft [8]. Penile cancer may also be identified by histology of usual type squamous cell carcinoma, papillary, warty/condylomas, basaloid, verrucous, or sarcomatoid [15]. HPV has been identified in 33–48% of penile tumors, with HPV 16 and 18 being the most frequently identified, though variations have been found within different histologic subtypes, with basaloid/warty squamous cell carcinoma types mostly associated with HPV infection [8,15]. HPV positivity has been associated with highly variable survival rates, with one study demonstrating improved overall 5-year survival, another showing no difference in 10-year survival, while a third showed worse overall survival for patients who were p53 and HPV positive [8,15].

## 6. Anal Cancer

There are 27,000 new cases of anal cancer per year worldwide, with a case ratio of women to men of 5 to 1 [9]. More than 90% of anal cancers are linked to HPV, making prevention with vaccination a major public health priority [9]. Anal intraepithelial neoplasia (AIN) denotes precancerous lesions graded I–III on severity of dysplasia [9]. Progression of precancerous lesions to cancer is variable, with patients infected with Human Immunodeficiency Virus (HIV) at the highest risk of cancer progression due to the HIV impact on CD4 cells and immune response against HPV [9]. Screening with anal pap-smears has not been found to be particularly helpful as within HIV-positive patients, being at the highest risk, 67% are HPV-positive, leading to over-testing [9]. Anal cancer can present as a variety of symptoms, from fissures, hemorrhoids, incontinence, pain, or itching, or may be asymptomatic [9]. HPV positivity within the tumor is generally associated with improved overall survival; however, more than one-third of anal cancer patients die within five years of diagnosis, with a 5-year case-to-death ratio of 86% for women and 89% for men [4,8,16]. While there may be advantages to screening at-risk populations (i.e., men who have sex with men) for anal HPV disease, no current guidelines exist for this screening, and screening efficacy remains a matter of debate [19].

## 7. Oropharyngeal Cancer

Incidence of oropharyngeal cancer is three times higher for males than females [10]. In the United States, 12,600 cases were reported from 2012–2016 and over 70% of oropharyngeal cancers have been associated with HPV [10,11]. Early signs of disease include neck swelling, sore throat, or dysphagia [10]. Tumors of the head and neck are classified as HPV positive or HPV negative, and most commonly occur at the base of the tongue or palatine tonsil [10]. Men and women who have sex with men are at highest risk, as men pass HPV to their sexual partners, with risk highest in the immunocompromised and those with uncircumcised partners [1]. Oropharyngeal cancer has a case-to-death ratio of 75% in five years [4]. When compared to other causes of oropharyngeal cancer, those with HPV-positive tumors have been demonstrated improved 3-year survival [8].

## 8. Cervical Cancer

Cervical cancer is the second most common cancer in women worldwide with a case-to-death ratio of 24% in 5 years, and over 340,000 deaths worldwide in 2020 [4,20,21]. HPV is the most common cause of cervical cancer, with infection of high-risk genotypes bearing the greatest risk of progression to cancer. This includes genotypes 16, 18, 26, 31, 33, 35, 39, 45, 51–53, 56, 58, 59, 66, 68, 70, 73, and 82 [22]. High-risk HPV subtypes are more common in developing countries than in the United States and Europe, with prevalence of high-risk types other than 16 and 18 in these developing regions [1]. HPV impacts cervical cells in a variety of ways as detected by cervical cytology, including atypical squamous cells of undetermined significance (ASC-US), atypical squamous cells high-risk (ASC-H), atypical glandular cells (AGC), LSIL, and HSIL. Pre-malignant changes may also be detected by colposcopic-driven biopsy or endocervical curettage in the form of cervical intraepithelial neoplasia (CIN) graded on a scale of I-III or adenocarcinoma in-situ (AIS). The incidence of cervical cancer experiences a bimodal distribution by age, with high rates of diagnosis for women aged 35–40 years and 65–80 years [4,20]. Prognosis for cervical cancer patients is dependent on stage of disease, presence of parametrial involvement, vascular involvement, and mode of metastasis, whether hematogenous or lymphatic spread, where those with lymphatic spread have poorer prognosis [23,24].

### Cervical Cancer Screening

Cervical cancer screening varies around the world, with the United States following the most complex algorithm [25]. Screening is completed by cervical cytology and HPV genotyping. The American Cancer Society screening guidelines released in 2020 recommend screening with cytology and genotyping every five years or cytology alone every three years beginning at age 25 and continuing through age 65 [26]. This is a change in recommendation from the 2012 American Cancer Society guidelines, which initiated screening at age 21, with rationale for the change being low cancer incidence in young populations and concerns of adverse obstetric outcomes due to overtreatment of abnormal cytology [26]. While these guidelines do not yet take HPV vaccination status directly into account, screening recommendations may change to consider vaccination status in the future. Despite testing availability, an estimated 14% of women in the United states are never screened [27]. In 2017, Australia began primary HPV testing, which has been a significant factor in the observed decrease in cervical cancer cases for the country [28]. Screening in itself is not yet available in many countries given barriers of cost, access to healthcare, and patient loss to follow up. Given this, countries have taken a variety of approaches such as screening at different ages or after the onset of sexual activity with cytology and genotyping, screening with colposcopy alone, or risk based assessment given presence of HPV-16 or -18 [25].

## 9. Virology and Vaccine Development

Given the prevalence and severity of the impact of HPV, global HPV vaccination has become a leading public health priority. The HPV genome encodes early and late proteins involved in viral replication, with two late proteins making up the viral capsid as the main interest in vaccine development. The HPV L1 capsid protein consists of variable and constant regions, with constant regions being specific to the HPV genotype and having high activation of host immune system through the self-formation of virus like particles (VLPs) [1,22]. Coat proteins from the capsid are displayed to immune system, leading to B cell activation and production of antibodies that are high affinity to the HPV specific L1 protein [22]. Epitopes then enhance T cell activation leading to production of cytokines and further activation of B cells, T cells, and macrophages, and thus the systemic immune response [22]. This approach to vaccine development and immune system activation is being adapted to other viruses and vaccinations, such as hepatitis B vaccines [22].

### 9.1. Initial Vaccines

Existing vaccines using the immunogenic response to VLPs include the bivalent, quadrivalent, and nonvalent vaccines. Bivalent vaccine constructed using L1 surface antigen of HPV-16 and -18 has been found to be cross protective against HPV 31, 33, and 45, even with just one dose of the vaccine, in addition to reducing genital warts caused by HPV 6 and 11 [4,29,30]. This protection with one dose of the bivalent vaccine has been found to be effective for up to 7 years [29]. The three-dose vaccine has been found to be effective for up to 11 years after administration, though after six and a half years, cross protection against other genotypes is no longer significant [4,29]. Furthermore, 9–14-year-olds receiving bivalent vaccination were found to have non-inferior responses after two doses at 0 and 6 months compared to those receiving three doses of the quadrivalent vaccine at 0, 2, and 6 months [30]. Adding low-risk subtype specific immunity, the quadrivalent vaccine conveyed protection against genotypes 6, 11, 16, and 18 [4]. Vaccination titers from both the quadrivalent and bivalent vaccines have been found to be 2–3 times higher than titers induced by natural infection [30]. A metanalysis completed in 2019 included data from 18 studies and found that women have higher antibody titer levels against HPV following vaccination than do men [31]. A systematic review of HPV vaccination effectiveness by number of doses administered looked at fourteen articles reviewing the bivalent or quadrivalent HPV vaccines [32]. All fourteen articles found significant efficacy at three doses, eleven found significant efficacy at two doses, and six found efficacy with one dose of the vaccine [32]. With the above studies in mind, the world health organization came to recommend two doses of the bivalent or quadrivalent vaccine in females age 9–15 years [30].

### 9.2. Newest Vaccine

The nine-valent vaccine, added genotypes 33, 35, 45, 52, and 58, covering 19% more cervical cancers than the quadrivalent vaccine [4]. In a systematic review of vaccine effectiveness in male populations, vaccination was found to be highly effective in HPV naive individuals and moderately effective at preventing high-grade anal intraepithelial lesions and persistent anal HPV infection, though many studies did not take into account the impact of adding vaccinated males to vaccinated female populations [33]. Studies have demonstrated that three doses of the nine-valent vaccine led to adequate levels of antibodies against all nine genotypes [4]. The nine-valent vaccine when directly compared to its quadrivalent predecessor was found to result in non-inferior titer levels of the HPV genotypes covered in the quadrivalent vaccine [4,30]. In fact, the nine-valent vaccine has been shown to have the best rates of prevention for CIN I, II, and III when compared to its predecessors [19]. Given this non-inferiority, the two-dose schedule of the nine-valent vaccine can be extrapolated from the success of the original quadrivalent vaccine. The nine-valent vaccine has been shown to cover more cervical cancers and be better at preventing pre-cancerous lesions [4]. While thought to be most efficacious in HPV naïve individuals, there is some thought that vaccination may prevent infection recurrence in those who clear the infection spontaneously, as most do [3]. Although the nine-valent vaccine has not expanded on the quadrivalent vaccine’s genotype specific coverage of benign cutaneous lesions, due to minimal health risk of benign cutaneous warts other than cosmetic appearance, cross coverage of the low risk HPV types have been hypothesized [1,2,17].

### 9.3. Vaccine Mechanism of Action

While this approach to vaccine development with the L1 protein has been successful, the development of vaccination against a variety of HPV genotypes has proven difficult as the L1 protein is not conserved among HPV genotypes, the L2 protein is conserved but cannot form VLPs [22]. Antibodies against HPV L2 proteins can protect against diverse HPV genotypes; however, antibody titers against L2 proteins are exceptionally low, making vaccine development challenging [22]. Vaccination against diverse genotypes would not only have utility in prevention on cancer and precancerous lesions, but L2 protein vaccination by VLPs would allow the prevention of a variety of benign cutaneous lesions, decreasing public health burden of cutaneous warts [34,35]. Specifically, a chimeric L2 vaccine has been investigated using a peptide portion of the L1 protein of HPV-16 to enhance the responsiveness of the immune system to the L2 particle, enabling broader vaccination coverage [34]. A variety of other methods have been instituted to make L2 vaccines more immunogenic, with the nanoparticle vaccine showing the most promise, with protection against 14 oncogenic HPV types [22,36]. VLPs stability is limited by temperature, making cold chain preservation essential for vaccine distribution, limiting current vaccine distribution worldwide [36].

## 10. Vaccination Distribution and Acceptance

### 10.1. High Income Countries

As we look to expand vaccination and screening coverage globally, especially to less developed countries, the examination of vaccination rates in those with access to the vaccine gives us perspective on the future. Especially in the United States, vaccination uptake has been slower than other countries with access to vaccination [37]. Vaccination within the United States increases with birth year, with vaccination by age 13 increasing from 19.9% of those born in 1998 to 62.6% of those born in 2006 [38]. One study of women in the United States found that only 29.8% believed the HPV vaccine to be effective in preventing cervical cancer, and approximately two-thirds (63.6%) stated they did not know if the vaccine was effective, with an increase of not knowing if the vaccination is effective in non-Hispanic black American women [39]. Additionally, vaccination rates are low in women with lower levels of education and women who were unaware of recommendation for vaccination by a healthcare provider [39]. Women younger than 65 were more likely to believe that the vaccine was not successful [39]. Despite concerns raised at the time of vaccination approval, studies have found no association between infertility and receipt of the vaccine [40].

Uptake of the vaccination has been a public health issue of concern since the vaccines inception, with a survey completed in 2016 noting approximately 87% of adolescents did not have parental support for receiving the vaccine and approximately 89% noting religious reasons for not receiving the vaccine [41]. Many believe this to be due to religious concerns of promiscuity following vaccination. Adolescent males, those from the Southern United States and those from high-income families, were also less likely to receive the vaccination [41]. The American Cancer Society has subsequently recommended that healthcare providers focus discussions with parents on cancer prevention rather than sexual transmission [37]. A cross-sectional survey of parents completed in 2017–2018 found that safety concerns was the most common reason parents did not initiate or plan to initiate the vaccine series for their adolescent children [42]. Overall, recommendation of vaccination by a healthcare provider has been noted as an important mediator in vaccine initiation and series completion [42].

Meanwhile, Australian vaccination rates, distribution, and acceptance serves as a great model for uptake of vaccination. Quadrivalent vaccine distribution was originally nationally funded for adolescent females in 2007 and extended to males in 2013 [43]. This comes after implementation Australian national screening program in 1991 [43]. Studies of populations in Australia have found decreased prevalence of high-grade cervical anomalies in vaccinated populations [43]. With a change from cytologic screening to HPV DNA testing and addition of the nine-valent vaccination to vaccination schedules, a decrease in both cervical cancer incidence and mortality is expected, nationally [43]. While many countries face individual and unique barriers to vaccination, the success seen in Australia provides a model not only for distribution, but potential reduction in cancer incidence, morbidity, and mortality if similar high rates of vaccination can be globally achieved.

### 10.2. Low and Middle Income Countries

Vaccination programs for low- and middle-income countries have lagged substantially behind programs in high-income countries, with program approval for funding of national distribution being reached in 2011, reaching mostly higher-middle-income countries [44]. These countries are important targets of vaccination from a public-health perspective given incidence, morbidity, and mortality associated with HPV infection within them. The highest HPV infection rates in the world are found in Asian regions followed by Eastern Africa; women in low- to middle-income countries have a 42.2% infection rate compared to 22.6% of women in higher-income countries [1]. Given barriers to program approval and difficulties with global vaccination supply, lower-income countries did not receive support until 2017 [44]. Largely, programs in these countries have focused on the vaccination of girls age 9–11 as they are accessible with high rates of school enrollment and are not expected to have yet made their sexual debut [44]. Given controversies experienced in other countries, newly implemented programs have teams dedicated to identifying and dispelling rumors associated with the vaccine [44].

A large barrier to vaccination in these countries is the need for refrigeration of the vaccine and cold-chain supply. This may be solved by lyophilized formulation or with heat-stable capsomer preparations. Lyophilization involves dehydration of vaccine components, then frozen in powder form for transfer at higher temperatures. Heat-stable capsomer preparations would allow for a greater degree in temperature fluctuation as well, allowing for decreased transport cost and increased accessibility. These formulations are not currently available for HPV vaccines [30].

## 11. Vaccination Impact

Despite barriers to global vaccine distribution, success of the vaccine and public health implications on a global level should not be discounted. High and sustained levels of vaccination have been associated with significant reduction in high-risk HPV positive infection, in addition to associated cervical pathology, resulting in significant herd immunity for the male population [30]. In Australia, a significant decline in anogenital warts in unvaccinated males has been attributed to high vaccination rates in females [43]. A vaccination study in men showed no development of penile cancer or PeIN in those who received the vaccine [8]. An estimation of vaccination impact using the bivalent and nine-valent vaccines as models estimated a lifetime risk of CIN3+ at 1.2% and 0.5%, respectively, assuming cross-protection and lifetime efficacy of vaccination [45].

Models estimating elimination or substantial reduction in cervical cancer cases attributable to HPV take into account rates of vaccination in addition to screening. A modeling study of the United States completed in 2020 estimated cases could decrease to 4 new cases per 100,000 woman-years by 2038–2046 [27]. In this model, elimination was more readily achieved with an increase in screening to 90% of the population than with increase in vaccination to 90% of the population (if achieved at 90% by the year 2020 [27]. Adding in 90% vaccination of the male and female population compared with 90% vaccination of the female population did not change the year by which their models predicted 4 new cases per 100,000 woman-years, while 90% screening predicted this incidence of cervical cancer by 2028–2033 [27]. While the aforementioned model is specific to cervical cancer, models like this demonstrate the importance of screening in combination with vaccination to achieve a reduction in disease pathology and emphasize the importance of global vaccination and screening. Meanwhile, a similar modeling study of Australia estimated 4 cases per 100,000 woman-years by 2028 if high rates of screening and vaccination are maintained nationwide [28].

## 12. Vaccination Schedules

Recommendations for the timing of vaccine administration have varied since vaccine approval in 2007. Vaccination for men 9–26 years was approved by the CDC in 2009. In 2019, the United States Advisory Committee on Immunization Practices guidelines changed to include vaccination catch up for those up to age 26, and shared decision making for adults up to age 45 who have not had previous adequate vaccination, with a focus on those at risk for exposure [37]. The American Cancer Society recommendations released in 2020 qualify these recommendations, as vaccination starting in early adolescence offers maximum protection and public health benefit, with vaccination of all 9–12-year-old children having the potential to prevent 90% of cancers caused by HPV, while catch-up vaccination in those up to age 45 has limited public health impact [37].

## 13. Future of HPV Vaccination

Although efficacy of current vaccines is well established, the potential for improvement exists in therapeutic potential of vaccines, increased HPV genotype coverage, and elimination of cold-chain supply. Both L1- and L2-based vaccines would have no curative or therapeutic intent, as they would not impact viral DNA that has already integrated itself into the host genome. In studies of those with cancer, higher rates of T-cell response are associated with improved clinical outcomes [3].

Given the success of prophylactic vaccination, therapeutic vaccination, namely against proto-oncogenes E6 and E7, is an area of great interest. E6 impacts the p53 pathway, and E7 acts through the retinoblastoma (Rb) tumor suppressor gene ultimately leading to increased p16^INK4a^ expression, a pathway attributed to dysplastic changes from HPV in many epithelial tissues [6,8]. Therapeutic vaccines are in development with the use of *listeria* monocytogenes as a vector to drive immune response against the E7 proto-oncogene for treatment of cervical cancer [3]. Goals of this method is to increase the immune response as mediated by CD4+ and CD8+ T-cells [19]. This approach is very similar to that of peptide vaccines used to treat vulvar and vaginal cancer, also using immune response against the E6 and E7 proto-oncogenes [3]. Viral vectors have also been investigated as a means to activate the T-cell medicated immune response against E6 and E7 [19]. Others still include synthetic plasmids targeted against E6 and E7 [3]. A potential therapy investigates targeting these proto-oncogenes through the CRISPR/Cas9 system, with main concern for these therapies being off-stream effects [30,46].

Single-chain variable antibody fragments (scFvs) are another area of interest in anti-tumor management, especially with respect to HPV. These antibodies can be engineered for expression intracellularly, or to target cells directly [47]. Specifically, scFvs have been identified with the ability to bind to epitopes of oncoproteins expressed by HPV-16 (16E7) and interfere with protein function [47]. Other scFvs have been identified that could work by interfering with E7 binding to the pRb tumor suppressor directly [47]. A third scFv, which has already demonstrated anti-tumor activity, works by binding cassein kinase II and does not exhibit anti-E7 properties directly [47]. All three mechanisms or scFv action could be used individually or in combination to target tumor activity for therapeutic effect.

## 14. Lessons Learned: Applications to the COVID-19 Pandemic

While causing very different clinical manifestations, long-term sequelae, and global implications, lessons learned from HPV and vaccination for the prevention of disease may be applied to SARs-CoV-2 vaccination and prevention. Current challenges in SARs-CoV-2 vaccine administration mirror those of HPV vaccination, as cold-chain supply greatly limits both vaccines. Cold-chain supply alone contributes to up to 80% of vaccine costs [48]. Lyophilized powder would be ideal for both vaccinations, allowing for transport as powder, resuspension, then injection or intranasal administration in powder form [48]. With the improvement in vaccine stabilization, leading to a decrease in vaccination cost, improved global vaccination rates may be reached.

## 15. Conclusions

HPV and its associated pathologies will continue to be a key focus in public health given the range and severity of disease associated with infection. Despite the development of vaccination for the prevention of disease, a significant global burden of these pathologies still exists. HPV infection can lead to a variety of both benign and malignant pathologies. Vaccination against HPV is the only current vaccination available for prevention of cancer. Vaccination against HPV faces a variety of challenges worldwide, similar challenges faced by vaccination against SARs-CoV-2 including heat stabilization of the vaccine product and cold-chain supply. Many improvements to vaccination for the prevention and treatment of HPV are being investigated, though they are likely to face similar barriers as current HPV vaccines. While programs for vaccination continue to expand and reach development, public buy-in to vaccination presents culture-specific barriers, and programs targeting misinformation and public concerns remain necessary to improve vaccination compliance. Healthcare providers play an important role in vaccination goals, as many patient’s value healthcare providers’ recommendations. Until high rates of global vaccination and immunity to the plethora of HPV genotypes leading to both benign and malignant processes can be achieved, we will continue to face both clinical and costly outcomes of this virus.

## Figures and Tables

**Table 1:** Summary of non-cervical HPV related malignancies.

Malignancy	Prevalence/Incidence	Association with HPV	Prognosis of HPV Positive Disease
Vulvar	3–5% of gynecologic ^†^	Direct for some disease, 25–43% ^†^	Higher overall survival and disease free survival ^€^
Vaginal	1–2% of gynecologic ^‡^	Direct for some disease ^‡^	Overall survival 35–85% ^ψ^
Penile	0.8–4.4/100,000 ^¥^	Direct for some disease ^¥^, 33–48% ^Φ^	Highly variable ^¥,Φ^
Anal	27,000/year ^£^	>90% ^£^	Improved overall survival
Oropharyngeal	12,600 from 2012 to 2016 ^₤^	>70% ^₤,§^	Improved 3 year survival ^¥,₡^

^†^ [6], ^‡^ [7], ^¥^ [8], ^£^ [9], ^₤^ [10], ^§^ [11], ^€^ [12,13], ^ψ^ [7,14], ^Φ^ [15], ^₡^ [4,16]. Human Papilloma Virus (HPV).
